# Functional Chitosan and Its Derivative-Related Drug Delivery Systems for Nano-Therapy: Recent Advances

**DOI:** 10.3390/pharmaceutics16030337

**Published:** 2024-02-28

**Authors:** Zixu Wang, Fangying Yu, Fuqiang Hu

**Affiliations:** 1 College of Pharmaceutical Science, Zhejiang University, Hangzhou 310058, China; 22119095@zju.edu.cn; 2 National Key Laboratory of Advanced Drug Delivery and Release Systems, Zhejiang University, Hangzhou 310058, China; 3 Department of Diagnostic Ultrasound and Echocardiography, Sir Run Run Shaw Hospital, School of Medicine, Zhejiang University, Hangzhou 310016, China; hzyfy@zju.edu.cn

**Keywords:** chitosan-based polymetric materials, nano-therapy, design criteria, preparation methods, biomedical applications

## Abstract

In the struggle against diseases, the development of nano-therapy has certainly been a tremendous progression owing to the various superiority, and chitosan is no doubt a kind of prominent biopolymer material with versatility for applications in disease treatments. For the rational construction of chitosan-related nano-biodevices, it is necessary to pay full attention to the material itself, where it is the material properties that guide the design criteria. Additionally, the well-matched preparation methods between material carriers and therapeutic agents draw much attention to the final construction since they seem to be more realistic. In detail, we present a comprehensive overview of recent advances in rational construction of chitosan-related nano-therapies with respect to material-property-oriented design criteria and preparation methods in the current review article, based on the foundation of continuous investigations. Based on this review, a portion of the various uses of chitosan-related nano-biodevices for biomedical applications are specifically discussed. Here, the strategies demonstrate the versatility of chitosan well, and the concept of being simple yet effective is well illustrated and vividly communicated. Altogether, a fresh concept concerning multi-functional chitosan and its derivative-related drug delivery systems for nano-therapy is proposed in this review, and this could be applied to other materials, which seems to be a novel angle.

## 1. Introduction

Nano-therapy has flourished recently due to its inspiring and unique properties in treatments for diseases, such as improving solubility, lengthening the in vivo circulation time of free drugs, crossing biological barriers, high disease target site selectivity, space/time control release, combination therapies overcoming drug resistance, and usages for novel imaging techniques [1], which profits from disease and material property-oriented design and developments of materials. In a quest for seeking and establishing significant nano-devices in biomedical applications and conquering innumerable extreme hardships encountered in disease treatments, biodegradable polymers have gradually captured the attention of investigators. The natural polysaccharide macromolecule is one of the most widely used materials for nanomedicine, among which chitosan is an indispensable part [2].

Chitosan, obtained by deacetylation of chitin, which primarily originates from shells of crustaceans, insect shells, as well as certain fungi, is a natural cationic polysaccharide owing to its manageable and active amino groups [3]. Actually, chitosan is the degraded product of chitin through acid hydrolysis, enzymatic degradation, or both, and it is substantially abundant in the natural world. Being a natural product, chitosan is highly safe compared to many other polymer materials, and it is beneficial due to the biodegradable nature of its structure and superiority of natural products, and the U.S. Food and Drug Administration (U.S. FDA) has recognized chitosan as Generally Recognized as Safe (GRAS) and approved it as a food additive [4]. More importantly, an increasing number of studies have focused on the biosafety of chitosan and its derivatives [5,6], making sense of the progress in this aspect. Similar to other synthetic or biopolymers, chitosan is characterized by a series of properties, including structural units, degrees of polymerization, and accordingly, molecular weight, which have been repeatedly confirmed and verified in various theories and studies; however, the attraction of chitosan extends far beyond these properties [7]. Compared to other polymer materials, the cationic polysaccharide structure endows chitosan with a variety of properties, such as biocompatibility, cationic nature, targeting ability, and so on [8,9]. Fortunately, these features are suitable for some disease treatments, and the origin is common. Considering its fascinating physiochemical properties and biological activity, chitosan has long been integrated into the construction of nano-biodevices for biomedical treatment processes. Not limited to this, it is imperative to note that sometimes, the structural modifications of chitosan, defined as chitosan derivatives, are of great significance for expansion, which means every daring attempt aims to achieve formulations with more promising and versatile characteristics [10]. Essentially, this is part of chitosan’s coverage for maximum functionality. 

Most recently, plenty of works have reinforced and validated the plasticity of multifunctional chitosan and its derivative-related drug delivery systems for nano-therapy, which derives from the theory of structure determining the nature [11,12,13]. Parallel to the growing interest in natural biopolymers, this field has become more attractive. With the rapid increase of chitosan-based nano-therapies, reflections on innovation have come into prominence. Generally, every design is distinctly deliberated with multiple elements owing to the consensus that the selection of material serves a function; thus, giving rise to a straightforward, comprehensive, but reasonable system. Here, chitosan is of particular significance for biomedical applications with respect to both the disease and the material itself, which constitutes the rational design in combination with preparation methods’ advances, conducive to innovations in chitosan-based biomedical nanodevices for diverse diseases. 

**Figure 1:**
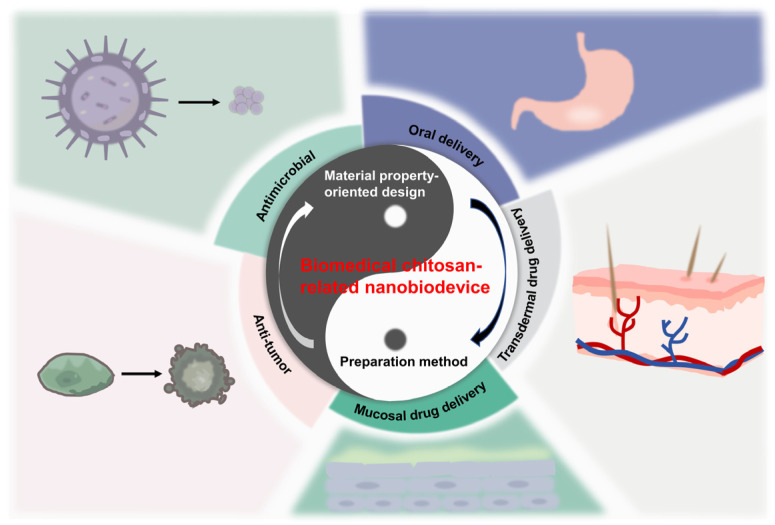
Schematic illustration of rational construction of functional chitosan and its derivative-related drug delivery systems for nano-therapy.

We present a holistic description of recent advances in the rational construction of chitosan-related nano-therapies with respect to material property-oriented design and preparation methods in the current review article, based on the foundation of continuous research, as exhibited in Figure 1. Based on this, the various uses of chitosan-related nano-biodevices for the treatment of diseases are carefully discussed. Every overview and summarization could bring about renewed insight into the following design. 

## 2. Advances in Rational Construction of Chitosan-Based Nano-Therapies

**Figure 2:**
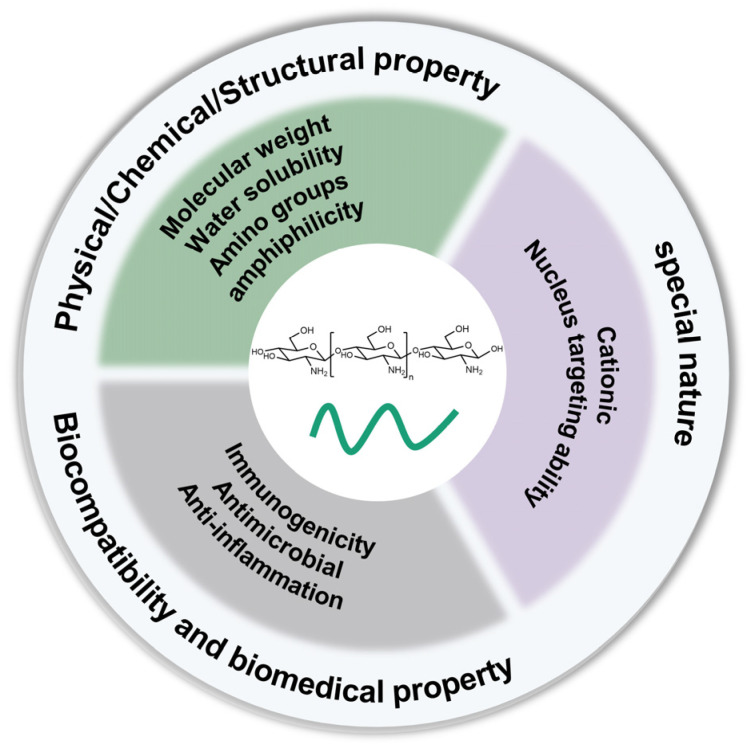
Molecular structure of chitosan and main material property-oriented design criteria of chitosan-based nano-therapy.

Chitosan has been explored for treatments of diverse diseases for decades, and researchers have been committed to combining it with nano-therapies to give full play to the advantages of chitosan. Based on grounded demand, it is of necessity to pay full attention to the material itself, and here, the material property-oriented design criteria are carefully discussed. Furthermore, the final construction profiles lean toward the well-matched preparation methods between the material carrier and the therapeutic agents, which seems to be another pivotal aspect. Much progress has been achieved on the rational construction of chitosan-based nanoparticles, mainly referring to the design criteria (Figure 2) and preparation methods of these nanoparticles, and they deserve further discussion.

### 2.1. Material Property-Oriented Design Criteria

#### 2.1.1. Amino Groups

The most intuitive and essential property of polymer materials is definitely the molecular weight. The molecular weight of chitosan is always the first physiochemical property to be considered for both preparation and utilization since it is in profound and extensive relation with other properties. Meanwhile, researchers put emphasis on molecular weight when designing multi-functional chitosan and its derivative-related drug delivery systems for comprehensive applications in many instances. For the production of chitosan, their primary amino groups are generated during the deacetylation process. Alterations of molecular weight directly bring about a changeable number of modifiable amino groups, leaving us with the first consideration for it. Due to the necessity to modify amino groups for functional usages, various structural modifications have been accomplished on chitosan through certain chemical reactions with the versatility of these amino groups, commonly including grafting reactions and chemical crosslinking strategies [14]. The modifications confer chitosan with amelioration of the properties for diverse biomedical applications. Even more, the number of amino groups also impacts the adsorption capacity of chitosan for proteins in a non-covalent manner, distinguished from chemical joining [15]. It can be pretty much assumed that the molecular weight of chitosan is indicative of many kinds of characteristics through altering the number of amino groups. 

#### 2.1.2. Water Solubility and Amphiphilicity

Apart from the number of amino groups, water solubility and amphiphilicity as related issues have to be mentioned, which often jointly occur. The water solubility of chitosan with different molecular weights and degrees of deacetylation varies, thus meaning they dictate the suitability of chitosan. Most definitely, the production of chitosan oligosaccharide, a derivative of chitosan with a degree of polymerization that is less than 20 and a molecular weight lower than 3.9 kDa, by either chemical or enzymatic hydrolysis, endows it with highly versatile biological activities and, especially, remarkable water-soluble properties [16,17]. The resultant biocompatible polymers with reduced toxicity implement a pro-absorptive mechanism by reconstructing the physical structure of the intestinal mucus layer to improve the utilization efficiency of water-soluble oral drugs for types of gastrointestinal-related irritations and diseases [18]. 

Due to the water insolubility of chitosan, its appeal is limited, and improvements such as chemical modification of chitosan are often performed prior to further utilization. For the purpose of enhancing solubility, three different molecular weights of chitosan were modified by using p-Coumaric acid. The obtained chitosan derivatives partially enhanced the water solubility over a wide range of pH values and antioxidant properties, although these properties decreased as the molecular weight of the corresponding native chitosan increased [19]. As a water-soluble restricted polysaccharide, efforts have never ceased for optimization, and a usual strategy is the selective oxidation mediated by 2,2,6,6-tetra-methyl-1-piperidinidyloxy (TEMPO) using laccase as a co-oxidant, displaying increased solubility and decreased viscosity in solution [20]. Productively, this kind of design brought about a pH-responsive chitosan hydrogel stabilized by self-crosslinking of the obtained dynamic imine bonds. In view of the aforementioned chemical methods of modification, a physical method was adopted, modulating the concentration of the solution and varying the precipitants. The treated chitosan became soluble in acid-free water, and its solubility was up to 8.02 mg/mL [21]. For chitosan, the performance of water solubility displayed unprecedented significance.

Practically, chemical modifications imparting alternations in water solubility tend to yield amphiphilic chitosan derivatives, opening new perspectives toward chitosan and facilitating drug delivery. Within the drug delivery vehicles, there has been increasing focus on self-assembly nanostructures of biopolymers, with amphiphilic chitosan derivatives being typical. Accumulated references deal with amphiphilic modifications of chitosan for realistic usages, and these designs usually put emphasis on covalent grafting of functional hydrophobic modules onto the chitosan hydrophilic backbone via amide or esterification reactions for efficacious drug encapsulation and delivery, ranging from small-molecule drugs to proteins and peptides, with intravenous or oral administration [22,23,24,25]. Typically, it is the creation of micelles with core-shell nano-spherical structures through self-assembly in aqueous environments that is the general structure suitable for encapsulating drugs. Besides, a facile route of a two-step process toward amphiphilic hybrid molecular brushes (HMB) with a chitosan backbone and, concurrently, grafted chains of poly(acrylamide) and polystyrene, was developed, which was attributed to being amphiphilic in nature to adapt to the medium [26]. The design presented a brand-new possibility of amphiphilic chitosan derivatives beyond core-shell nanospheres.

Within these self-assembled structures, the hydrophobic and hydrophilic moieties are responsible for aggregation into micellar cores and kinetic stability to prevent precipitation of the system, respectively, when dispersed into external solution, and the resultant nanostructures can display distinct geometric patterns with adjustability and behave differently due to the intended design concerning moieties and solvent media [27]. From this perspective, it can be assumed that if the balance between hydrophobicity and hydrophilicity is disturbed, the stabilized constructs will depolymerize and offer structures with specific tailorable opportunities for treatments. For instance, when the photosensitizer, pheophorbide A (PhA), was conjugated to a water-soluble glycol chitosan (GC) through an ROS-sensitive thioketal (TK) linker, the amphiphilic GC–TK–PhA conjugates could arrange themselves into nanoparticles and remain photoinactive due to their clustering self-quenching effects. Therefore, upon ROS-sensitive TK bond cleaving in response to an ROS-rich tumor tissue microenvironment, PhA were released away from each other in a photoactive form, thus demonstrating good anti-tumor potential [28]. In another investigation, considering the unique metabolism of cancer cells (Warburg effect) and the target for interfering with glucose metabolism, an antiglycolytic amphiphilic polymer, GC-PBA, synthesized through chemical conjugation between GC and hydrophobic phenylboronic acid (PBA), was developed. Normally, GC-PBA derivatives could form stable micellar structures under physiological conditions and capture intracellular glucose on account of the selective complexing ability between PBA and glucose, resulting in energy deprivation and inhibition of tumor growth [29]. The stable micelle structure provided the ability of blood circulation and targeting, and changes in the intracellular glucose concentration gave rise to structural dissociation emphasized on hydrophilic–hydrophobic transition with resultant therapeutic effects. 

What can be envisaged is that amphiphilicity is a relative concept in which the balance between hydrophilic and hydrophobic can be seized for nano-therapies, whether building or breaking, making it attractive to remodel amphiphilicity through structural modifications. Far away from this, what is totally distinguished from micelle is the existence of coatings for delivery. Especially for gene therapy, nonviral nanocarriers with low immunogenicity and high efficiency of gene delivery remain formidably challenging, and a novel stimuli-responsive polysaccharide-enveloped liposome carrier was constructed by depositing redox-sensitive amphiphilic chitosan and hyaluronic acid layer-by-layer onto the liposome via covalent linkage and noncovalent interactions to trigger the sequential payload release [30]. Frequently, the strategy of liposome nanocarriers incorporating functional polysaccharide coatings is utilized [31]. From our exploration, we obtained insights into the underlying roles of amphiphilic chitosan derivatives for drug delivery, and it is clear that there are more possibilities no matter the structure or function.

#### 2.1.3. Cationic Nature

The rare cationic feature of chitosan in nature imparts specialized applications for drug delivery. One of the most classical usages based on charge adsorption is the formation of the polyelectrolyte composite micelle, prepared through polyelectrolyte self-assembly. For instance, lignosulfonate (LS), an anionic polymer with the components sulfonic acid and carboxyl and hydroxyl groups containing hydrophobic segments, was employed with chitosan to prepare chitosan/lignosulfonate composite micelles (CS-LS) through π–π and electrostatic interactions, displaying an average diameter of 239 nm [32]. With respect to the carrier design, chitosan-related nanocarriers facilitate delivery of specific agents, such as nucleic acid-loaded agents, due to the wealth of negative electric charges, and arduous efforts have been made by a myriad of scientists to seize the interaction [33,34]. Here, chitosan tends to be the ideal choice for burst co-delivery of siRNA and chemotherapeutics, alongside a further cationic modification with polyethyleneimine (PEI) to fetch a stronger electrostatic interaction than the naturally occurring polycationic chitosan [35]. Besides, Yu et al. aimed to condense negatively charged glucose oxidase onto chitosan through charge adsorption to construct a nano-cascade reactor, thus inspiring and exacerbating the following hypoxia cascade reaction for optimal synergistic anti-tumor potency [36]. It is pretty clear that the cationic nature makes sense in the delivery of negatively charged macromolecules and, sometimes, these amino groups could complex metal anions well [37]. The efficient delivery also calls for overcoming lysosomal sequestration, named endosomal escape, through the proton sponge effect, in which case the buffering capacity of chitosan was decently verified as to the cationic nature. Furthermore, it was revealed that chitosan possesses a higher buffering capacity than PEI in the endosomal pH range on a molar basis [38].

#### 2.1.4. Immunogenicity

Cumulative research has emphasized the immunogenicity of polysaccharide adjuvants, especially chitosan with vaccine adjuvant properties. In order to prevent mucosal infection, a complex and strict mucosal immune system evolved, which is the first line of defense against pathogen invasion and infection. As a result, a quaternized chitosan-based hydrogel was prepared as a nasal adjuvant of the H5N1 vaccine for the mucosal immune system, and the defined study demonstrated that the moderate quaternization degrees prolonged the antigen residence time in the nasal cavity, resulting in the most potent subsequent systemic humoral responses, as indicated by better enhancements in IgG titer [39]. This investigation of the structure–activity relationship between the quaternized chitosan-based hydrogel and the immune response made the effects of chitosan as an immune effector clear. 

While the mucosal immune system has an extensive and powerful role, the most comprehensive and integrated immune systems are humoral and cellular immunity, on which the design strategies of the chitosan-related immunological effector depend. Recently, Zhang et al. [40] constructed mannose-modified stearic acid-grafted chitosan (M-CS-SA) micelles dedicated to the activation of the cyclic GMP-AMP synthase (cGAS)-stimulator of interferon gene (STING) signaling pathway and the maturation of dendritic cells (DCs), giving rise to the roles of chitosan in building the anti-tumor cellular immune response. The combinatorial therapy of programmed administration of oxaliplatin and M-CS-SA effectively achieved a tumor inhibition rate of 76.31% and verified chitosan for its tangible application value in immunotherapy. For this reason, the potential of chitosan for extensively being used as a vaccine delivery vehicle was assessed [41]. It is clear that the immunogenicity of chitosan is not limited to such an extent, and many other properties are incorporated with it for further usages. 

#### 2.1.5. Nucleus-Targeting Ability

As is known, the size of nuclear pores is strictly controlled for agents entering and exiting the nucleus due to its essential role for cellular life activities. The nucleus-targeting ability is an ancient and rewarding issue of chitosan since the potential mechanism is uncertain, although the property is widely applied. In many cases, especially for the intracellular delivery of nuclear-targeted drugs, such as doxorubicin, chitosan was always preferred, and there was an explicit and intense co-localization signal between the nucleus and chitosan-based nanoparticles [42,43]. At the same time, the nuclear-targeting capability of chitosan could enhance the anti-tumor effects of lactoferrin on glioma [44]. It is widely acknowledged that chitosan nanoparticles’ size and the density of the nuclear localization sequence are relevant to the nuclear localization degree [45,46], while researchers are still wondering about the unique nucleus-targeting mechanism of chitosan independent of these factors.

#### 2.1.6. Antimicrobial Activity

For microbial pathogen infections, innovation in every generation of antibiotics aims to fight drug resistance and achieve better treatments for emerging microbial infections. However, it is a huge challenge. Chitosan is one of the most promising natural biopolymers possessing antimicrobial activity, and with deeper exploration, the antimicrobial features and applications of chitosan have gradually become clearer [47]. When it comes to microbial cells, the first to be mentioned refer to the cell wall and cell membrane, which are the parts of the cell that are in direct contact with the external environment. Undoubtedly, the cell wall or cell membrane do matter for various types of bacteria and fungi since they work to maintain cell morphology for support and protection from external environments, regulate the movements of substances into and out of the cells, and mediate intercellular communications. For bacteria, the cell wall of Gram-positive bacteria has a simple composition, in which a thick and dense layer of peptidoglycan embedded with teichoic acid is the main component, conferring a negative charge to bacteria. Likewise, the cell wall of Gram-negative bacteria is negatively charged, mainly attributed to the anionic components of lipopolysaccharides in the cell external membrane, although there is no teichoic acid in the cell wall [48]. Under these circumstances, the strongly positively charged chitosan have a natural attraction to the bacterial cell wall and membrane, presumably based on the electrostatic interactions since there are abundant amino groups on the polymerized long chains of chitosan, which can be protonated when the pH value of the aqueous medium is lower than its pK_a_ value, imparting a positive charge to chitosan. In this case, the cell permeability tends to be altered, and cell membrane lysis can occur, thus exerting an antimicrobial effect. 

Su et al. [49] designed and synthesized a kind of arginine-modified chitosan derivative, DAC, which was characterized by FTIR, ^1^H NMR, ^13^C NMR, DSC, and elemental analysis. Meanwhile, the antimicrobial activity of DAC was evaluated against both Gram-positive and Gram-negative bacteria, exhibiting improved bacteriostatic effects and no significant toxicological differences compared to chitosan. This was achieved through grafting arginine-containing guanidyl onto C6 groups of chitosan while maintaining the integrity of C2 amino groups with antibacterial activity. This research utilized the electrostatic interaction between the bacterial cell wall/membrane and chitosan and increased the positive charges of chitosan and protonation by chemical modification methods to create a better antimicrobial effect [49]. In another study, Wang et al. constructed a kind of chitosan-modified Fe_3_O_4_ (CS@Fe_3_O_4_) nanomaterial to fight against *Acinetobacter baumannii* (*A. baumannii*), which firstly profits from binding of the negatively charged bacteria to the protonated amino groups on the chitosan molecular chain under acidic conditions and, subsequently, the disruption of the cell. As a result, Fe_3_O_4_ nanozymes can easily enter bacterial cells and exert an antibacterial effect mediated through reactive oxygen species (ROS) production and inhibition of biofilm formation. Intriguingly, the nanoparticles displayed better antibacterial activity to drug-resistant bacteria than drug-sensitive ones [50]. Further, the structure–activity relationship for antibacterial chitosan carrying cationic and hydrophobic moieties was investigated. The authors synthesized four water-soluble derivatives of chitosan by direct modification of the 2-amino group in the biopolymer chain through highly regioselective methods. They confirmed that a cationic group in chitosan showed a broad spectrum of activity toward both Gram-positive and Gram-negative bacteria. Further, they concluded that the presence or absence of a hydrophilic/hydrophobic balance decides whether the chitosan polymer acts as a highly selective antimicrobial agent [51]. Namely, chitosan is more active on cell surfaces containing negatively charged substances on the basis of electrostatic interactions or other non-covalent interactions in terms of expansion. 

This mode of action can also be applied on anti-fungal treatments, primarily for chitosan-sensitive fungi containing negatively charged phospholipids on the plasma membrane, which leads to disintegration of fungal cell membranes. On this basis, fungi species can be divided into two groups: chitosan susceptible and chitosan resistant [52], in which case this mode of action is proposed not to occur in chitosan-resistant fungi. It is worth mentioning that in-depth studies into the interactions between chitosan and fungal cell membranes are scarce. Intriguingly, the absence of a putative glucose transporter in *U. maydis* resulted in resistance against chitosan [53]. Moreover, inhibiting ergosterol biosynthesis acts as a vital way for chitosan to disturb plasma membrane integrity, which is one of the most important components of fungal cell membranes [54], and the fact that ergosterol is a prominent site of action for chitosan is accepted as a plausible opinion, which represents the important correlation between chitosan and fungal cell membranes [55]. There is another scenario where some researchers revealed that the fluidity of the fungal cell membranes was another key factor determining the sensitivity to chitosan, and it also depended on the composition of plasma membranes, with polyunsaturated fatty acids in particular [56,57]. Hopefully, the developments of omics, such as proteomics, transcriptomics, and chemo-genomics, will allow for deeply studying the modification of fungal cell membrane composition, and its interactions with chitosan are becoming clearer.

When the microbial cell wall or cell membrane is disrupted, chitosan, especially the low-molecular-weight ones, can easily enter the cell and have profound impacts on essential substances and physiological processes for life activities, imparting entry effects. Sun et al. provided a brand-new insight into the antimicrobial mechanism of chitosan. In the study, they revealed that chitosan uptake is mediated by clathrin-dependent endocytosis, and the following cellular internalized chitosan plays an important role in its antimicrobial activity against fungal pathogens. This means the antimicrobial activity of chitosan is shaped by the combination of several attributes, which refer to not only the disintegration and deformity of cells but also chitosan inside the cytoplasm binding with its intracellular targets and impairing the physiological processes [58]. For one thing, the protonation of free amino groups of chitosan turns it into a polycation, easily binding with negatively charged phosphate groups in the chain of nucleic acids or other negatively charged cellular essential substances [59,60], not to mention that chitosan/DNA complex nanoparticles have been investigated for gene delivery vectors for decades [61,62,63,64]. Further, it was reported that chitosan oligosaccharide, which is a kind of oligosaccharide that is composed of two to ten N-acetylglucosamines linked by the β-1, 4-glycoside bond and obtained by hydrolysis or degradation of chitosan, restrained the mycelial growth and spore germination of *C. fimbriata*, aroused by decreasing the ergosterol content of cell membranes, inducing reactive oxygen species accumulation, Ca^2+^ homeostasis dysregulation, mitochondrial dysfunction, and meta-caspase activation, coupled with the occurrence of apoptosis. Namely, it was an induction of programmed cell death modalities that interfered with normal cellular physiological processes triggered by chitosan oligosaccharides [55]. Hydrophobic modification of chitosan oligosaccharides for mitochondrial damage is versatile [65]. Meanwhile, in a recent study, it was also reported that chitosan could disrupt normal cellular metabolism, leading to significantly enhanced reactive oxygen species accumulation and, eventually, apoptosis [66]. In another study, the authors analyzed changes in the expression profiles of *A. ochraceus* upon chitosan treatment by RNA sequencing and identified differentially expressed genes involved in ribosome biogenesis, glycerophospholipid metabolism, ether lipid metabolism, and steroid biosynthesis progress [67], which is further important evidence for chitosan’s antimicrobial activity.

#### 2.1.7. Anti-Inflammation Activity

Inflammation has relevance in appreciable amounts of diseases for both origins and processes in maintaining a non-resolving state. Somehow, microorganisms are closely linked to inflammation on account of the fact that humans are partly microbial and exist in a microbial environment, under which circumstances inflammation is a frequent presence by means of protection from the spread of infection and repair of damaged tissues [68]. From this perspective, inflammation can be defined as a protective immune response mounted by the innate immune system to harmful stimuli in large measure [69]. In this case, for chitosan, with antimicrobial activity, having broad applicability on anti-inflammatory treatments seems to be predictable. Persistent exploration of complicated mechanisms of non-resolving inflammation ensures countless encounters with chitosan for anti-inflammatory activity. 

Chitosan and nano-chitosan display salient functions in skin protection, regeneration, and repair when there is wound infection present, and for this purpose the minimization of the inflammation phase is promoted by the antimicrobial activity of chitosan [70]. Thus, it is not hard to comprehend the numerous attempts to combine antimicrobial and anti-inflammatory activities in the same chitosan-related drug delivery system, giving full play to the functions of chitosan [71,72,73]. Besides, what has to be recognized is that anti-inflammation and immune regulation are inextricably linked, as seen in a study that delved deeper into the treatments of ulcerative colitis from the perspective of modulating intestinal immunity. Specifically, chitosan inhibited the activity of pro-inflammatory cytokines IL-6, IL-1β, and TNF-α and upregulated anti-inflammatory cytokines IL-10 and IgG in serum to regulate immunoglobulin [74]. Further, chitosan of different molecular weights was found to have variable secretion of nitric oxide (NO) for anti-inflammatory effects with distinct mechanisms, such as variation in pro-inflammatory cytokine production, NO regulatory mechanisms, and binding receptors [74,75]. In continuation of the intentional modification of chitosan, four chitosan-based hydrogels via crosslinking interactions were successfully constructed. They identified differentiated selective inhibition effects toward the cyclooxygenase-2 (COX-2) enzyme of the four hydrogels in comparison with the typical nonsteroidal anti-inflammatory drug (NSAID) *Celecoxib* (IC_50_ 0.26 μg/mL), among which the best performance was IC_50_ 0.42 μg/mL [76]. Nonetheless, researchers have found chitosan to have rewarding anti-inflammatory activity and potential mechanisms.

### 2.2. Preparation Methods

**Figure 3:**
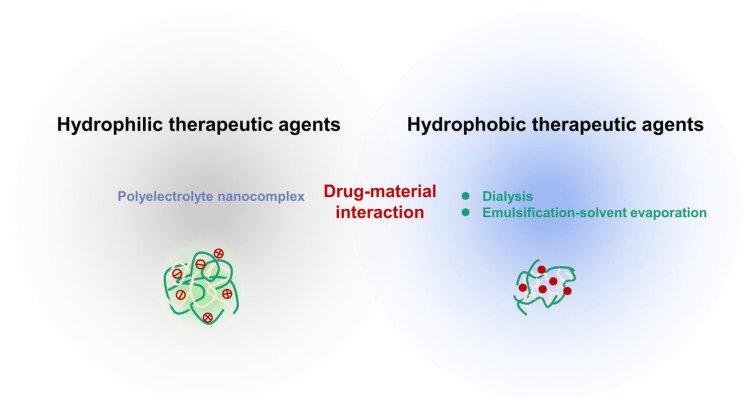
Preparation methods for rational construction of chitosan-based nanocarriers.

The rational construction of chitosan-based nanocarriers has been intended for drug delivery, initially categorized into hydrophilic and hydrophobic therapeutic agents. Actually, optimal encapsulation could primarily make the pharmacokinetic properties of drugs better, and they are used to exert synergistic effects in many instances, which are of great value for drug delivery. In further explorations, the nanocarriers could assist in the discovery of brand-new targets. To achieve the desired drug encapsulation effects, it is imperative to take multiple factors into account when selecting suitable drug loading methods, but overall, the water solubility of agents tends to be the paramount consideration [27], as depicted in Figure 3. 

#### 2.2.1. Hydrophilic Therapeutic Agents

With respect to totally hydrophilic drugs, the charge–absorption interaction tends to be a vital principle during the drug loading process since the cationic chitosan is renowned for its positive charge, and this is particularly well suited for macromolecules, such as proteins [36] and nucleic acids [77,78]. Under these circumstances, a polyelectrolyte nanocomplex is envisaged for desirable delivery forms in gene therapy, and the ideal transfection efficiency could depend on a series of formulation parameters, such as the molecular weight of chitosan, charge ratio of chitosan to DNA/siRNA (N/P ratio), chitosan salt form, DNA/siRNA concentration, pH, serum, and even routes of administration [34]. Usually, these factors are not single elements or simple combinations, which means when one formulation parameter is changed, the others will be restructured to obtain an optimal transfection efficiency [78]. 

There is no doubt that the chitosan-based nanocomplex brings about stability and protects against enzymatic hydrolysis to nucleic acids in the microenvironment. Sometimes, researchers contribute to encapsulating it into crosslinked hydrogels due to their biomimetic structures and properties. For example, Sun et al. prepared a near-infrared-responsive nanocomposite hydrogel, using methyl-methacrylate-modified chitosan as the macromolecular crosslinker, N-isopropylacrylamide as the backbone, and molybdenum disulfide nanosheets as the nanocomponents, via ultraviolet photopolymerization, to obtain enclosed polydeoxyribonucleotide, which served as an ideal wound dressing [79]. In another investigation, the precoating approach of protein corona was applied in chitosan-based nano-carriers for improving the therapeutic effects of nucleic acid drugs, as well as presenting a practical methodology [77]. A similar rationale occurs upon construction of siRNA-loaded chitosan nanoparticles, where the N/P ratio is calculated and precisely optimized over the simple complexation process; meanwhile, the combination of a crosslinker agent, such as sodium tripolyphosphate, and the above formulation could reinforce the ionic gelation interaction, counteracting many of the pitfalls associated with various uncertainties in the delivery system [80]. Collectively, the drug loading strategy of the polyelectrolyte complex leads to opportunities for hydrophilic drug delivery. 

#### 2.2.2. Hydrophobic Therapeutic Agents

Similar to hydrophilic drugs, chitosan-based nanocarriers facilitate the delivery of hydrophobic drugs with strong potential. It is widely acknowledged that the low solubility of hydrophobic therapeutic agents restricts further applications, which motivates the generation and innovation of drug delivery systems. Of course, an excellent nanocarrier has combined advantages and customized utilizations, yet besides these elaborated fabrications, improved solubility is always the original priority. Under the circumstances that chitosan-related nanocarriers are highly likely to contain hydrophilic and hydrophobic moieties at the same time for self-aggregation in aqueous solvents, the non-covalent interactions between hydrophobic drugs and hydrophobic units as the inner core could be the primary basis of encapsulation. In accordance with the classical theories of materials science and technology, polymer materials present a stretching state in their good solvents and display excellent solubility properties. By extension, every therapeutic agent to be encapsulated dissolves variably in different solvents and has its own good solvents, thereby providing a reference point for entrapping. Overall, it is the dialysis method and emulsification-solvent evaporation method that are categorized, depending on the procedure of removing organic solvents, and they are distinguished by volatility.

Accordingly, the selection of organic solvents is taken into consideration firstly among the many premises concerned. Most recently, Zeng et al. [81] prepared chitosan-based GSH-responsive glycolipid polymeric micelles to envelop doxorubicin (DOX) for chemotherapy treatment. In this regard, the dialysis method was adopted, where the prepared glycolipid polymers were dissolved in deionized water and stirred with DOX/DMSO solution for 2 h. The mixed solution was dialyzed to remove the DMSO solvent, and subsequently centrifuged to obtain a DOX-loaded micellar solution. The results showed that the encapsulation efficiency and drug loading of DOX were up to 69.6% and 12.2%, respectively, with superior in vitro release kinetic behavior. The high boiling point and nonvolatile nature of DMSO made it obvious to choose the dialysis method for removal. Meanwhile, they employed the solvent evaporation method to load immune checkpoint blockade (BMS-202) in similar chitosan-related glycolipid polymeric micelles. In detail, the BMS-202/chloroform solution was added dropwise to the micelle aqueous solutions (organic phase: aqueous phase = 3:10) with stirring, and the obtained mixed solution was sonicated. After that, the solution was stirred at room temperature overnight to evaporate chloroform, consequently acquiring BMS-202-loaded micelle aqueous solutions after centrifugation [82]. The in vitro drug release profile undergoes a cumulative 24 h release of 80%, without an apparent sudden release, laying the foundation for the subsequent in vivo anti-tumor effects. For this procedure, it is simple enough to select the evaporation method for the removal of chloroform since it is extremely volatile.

At the very beginning, the ratio of organic phase to aqueous phase is elaborately designed for the successful formulation of oil-in-water (o/w) emulsion after sonicating the mixture solution, and this ratio could be differentiated for different agents. Zhou et al. designed and prepared JQ1-loaded amphiphilic chitosan-based micelles via the o/w emulsion method for innovative anti-tumor therapy, where 0.3 mL of a chloroform solution of JQ1 was added dropwise into 10 mL of an aqueous solution of pre-formed micelle under stirring to form the o/w emulsion [83]. The process of removing chloroform was the same as that mentioned above within open atmosphere conditions. Here, the ratio of organic phase to aqueous phase was reduced with respect to the former, achieving 65.56% of encapsulation efficiency and 9.08% of drug loading, respectively. It can be hypothesized that the ratio of organic phase to aqueous phase, conditions of sonication, and even the concentration of agents have opportunities to impact the packaging of drugs, extensively and deeply, with which the quality of emulsions is associated. 

The choice of solvents is manifold. As for the chemotherapeutic drug mitoxantrone (MIT), the solvent evaporation method was applied for enveloping as well. In brief, the prepared amphiphilic chitosan micelle (10 mg) and MIT (2 mg) were dissolved in methanol, and then the solution was added dropwise into deionized water with stirring for 6 h at room temperature [36]. Similarly, the rotary evaporation method was used to remove methanol, and the final drug-loaded nanoparticle was obtained by ultrafiltration. Although, most likely, the amphiphilic chitosan polymers were not dissolved well in methanol due to the high water-solubility of low-molecular-weight chitosan, the premixing process of micelle and MIT in methanol through vigorous stirring could be the key to the loading, which is the so-called one-step precipitation method. The encapsulation efficiency and drug loading were up to 86.19% and 16.37%, which were extremely superior. It could be assumed that the prioritization of organic solvents during the drug loading process relied on the nature of the drug to a large extent, and it represents varying philosophies of encapsulation and drug–nanocarrier structural interactions.

#### 2.2.3. More Considerations

There are numerous determinants during the process of drug loading. For this consideration, an investigation reported the encapsulation and elution of a hydrophobic drug, desmethoxycurcumin (DMC), as a molecular probe, with the carboxymethyl-hexanoyl chitosan (CHC), intended to evaluate the entrapping efficiency, CHC–DMC interaction, and nanostructure variation, with the probe being encapsulated and released from the CHC nanoparticles [84]. They validated the occurrence of twisting and shifting between DMC molecules in the CHC/DMC nanoparticles via a significant induced circular dichroism signal and the bathochromic shift in the UV-vis absorption spectra through intermolecular hydrogen bonding, evidencing the nano-structural evolution history of CHC/DMC nanoparticles from encapsulation to release. This nano-structural model provided a better understanding of the mechanisms involved upon drug loading at the microscale. Moreover, sustained-release dorzolamide-loaded chitosan-coated polycaprolactone nanoparticles (DRZ-CS-PCL-NPs) with the bio-adhesive property were constructed by the single-step emulsification technique and optimized via the three-factor three-level Box–Behnken design for ocular delivery, with the composition of polycaprolactone (60 mg), chitosan (0.6%), and polyvinyl alcohol (1.5%), thus showing a 72.48% ± 5.62% encapsulation efficiency [85]. Here, the PCL and DRZ were dissolved in dichloromethane to form the organic phase, emulsified, and evaporated with a condition of constant stirring at 1000 rpm for 6 h. 

Investigators seek decent methods, and every attempt provides insight into the successful construction of chitosan-related drug delivery systems. Therefore, we might suppose there is no distinction between these methods as superior or inferior, as every therapeutic agent to be loaded can match with its own most suitable method, with one minor tweak probably resulting in a dramatic difference. Along with those outlined above, new technologies and methodologies could also assist in these explorations. On the basis of a thorough and in-depth understanding of drug–carrier structural interactions, the innovation of drug loading methods might be more accessible. 

## 3. Biomedical Applications of Functional Chitosan and Its Derivative-Related Nano-Biodevices

**Figure 4:**
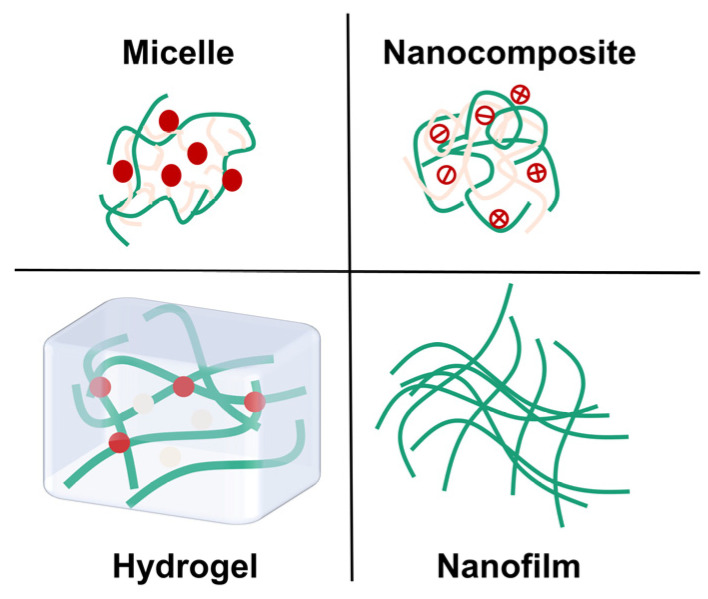
Different kinds of functional chitosan and its derivative-related nano-biodevices.

For a long time, until the present day, chitosan and its derivative-related nanoparticles are well-received for plenty of biomedical applications, which is a compelling demonstration of its potential in rational construction of nanomedicine delivery systems with different forms (Figure 4). As mentioned above, considerable progress has been made in the design criteria and preparation methods of rational constructions, and through this process it is important to be conscious of the wide range of chitosan applications. Chitosan shares a multitude of outstanding properties for both the nature of the material itself and the superiority for treatments of diseases, thereby enabling the well-designed nanoparticles to behave differently. This is no doubt of great benefit for nano-therapies since they offer structures with specific tailorable opportunities. Here, these opportunities are versatile, which means the properties are available on demand with combined applications. 

Upon designing functional chitosan and its derivative-related nano-biodevices, it is customary to take the majority of demands into consideration, and this correlates with the applicability and versatility (Table 1). The underlying logic is that when nanodrug delivery systems are designed, the diverse materials are cautiously selected since the characteristics of the material and structure guide the functions, and meanwhile, every design is carefully thought out. Based on the aforementioned description, it is obvious that chitosan is an ideal material for biomedical applications and worthy of being explored. Living up to expectations, for decades, researchers have not stopped exploring the multi-functional chitosan and its derivative-based nanodevices to realize the maximum functionality of chitosan. In this process, there is not a lack of exceptional and ingenious designs, and the applications in different diseases are well introduced in this section. 

### 3.1. Anti-Tumor

Among the numerous biomedical applications of chitosan, one of the most common usages is in anti-tumor treatment, and the concept of multifunctionality is implemented explicitly in this area. As concluded in a review, multifunctional chitosan-based nanoparticles have been extensively employed to combat multiple obstacles with cancer heterogeneity [86]. In a study conducted a couple of years ago, a DOX-encapsulated polymeric nanoparticle surface-decorated with chitosan was developed to increase the cytotoxicity of the DOX to tumor reinitiating cancer stem-like cells [87]. Here, the nanoparticle system could specifically target the CD44 receptors of target cells and programmatically release the DOX in acidic environments. Although the drug delivery system is rapidly developed nowadays and there is no shortage of unconventional designs, the multifunctionality of chitosan is well employed in this ideation for its modifiability, CD44 receptor targeting, positive charge favoring cellular uptake, and enzymatic degradation property. It is also worth mentioning that the amphiphilic copolymer PEG terminals of Pluronic F127 were partially activated, followed by oil-in-water emulsion and interfacial-crosslinking with chitosan in water, signifying that an emulsification-solvent evaporation method was used to develop the nanocarrier. In another study, researchers developed chitosan-coated, DOX-loaded, and aptamer-mesoporous silica nanoparticle (MSN) bioconjugates for breast cancer treatment, where chitosan and partial carboxylation chitosan were coated on MSNs via covalent crosslinking and chemical linkage [88]. The chitosan coating imparted pH-responsiveness and endo/lysosomal escape ability to MSNs, which was attributed to the abundance of protonatable amino groups on chitosan. It can be hypothesized that chitosan coating is frequently employed, while there is a combination of organic materials for constructing nanocarriers, which brings about more possibilities for drug delivery, and the rationale refers to the multifunctionality of chitosan. Importantly, this presumption was verified in a recent article. For the assembly of an in situ injectable thermo-responsive nanocomposite hydrogel with a calcium peroxide nanoparticles core, hydroxypropyl chitosan was first considered as a functional substance with adhesive hydroxyl groups to adhere to and grip tumor tissue, endorsing the modifiability and biocompatibility of chitosan [89].

Precisely because of the exceptional nature of chitosan, sometimes it was preferred to employ chitosan as the primary moiety in model and variable research. Sun et al. proposed and first fabricated a subsequent in situ culture strategy based on the developed chitosan nanoparticle cellular-compatible soft interface by electrospray, and subsequently the soft substrate was modified by the given substances on demand to endow it with the capability of specific capture of rare circulating tumor cells for isolation, purely and viably [90]. Here, chitosan is more likely utilized as an instrument due to the high expectations, where its modifiability, hydrophilicity, as well as biocompatibility are thoroughly recognized. What is more, chitosan is commonly selected for the fundamental fabrication of models in some variable and factor studies. To explore the effects of tumor microenvironments, especially for the enhanced permeability and retention (EPR) effect on nanoparticle-based targeted delivery systems, self-assembled glycol chitosan nanoparticles were prepared, and the near-infrared fluorescence (NIRF) dye Cy5.5 was labeled on them, aiming for comparative analyses using non-invasive NIRF imaging [91]. The authors conducted comprehensive studies on the correlation between the nanoparticle targeting efficiency and tumor microenvironment consisting of the extent of the intra-tumoral extracellular matrix, properties of angiogenic vessels inside of tumors, tumor pH, and intra-tumoral micro-vessels, where glycol chitosan provided substantial assistance due to its stable and available nature. 

As one of the most common and widespread diseases, tumors pose great threats to human lifespan and healthy living. For many nano-based therapies, chitosan and its derivate-related nano-biodevices have received considerable attention for research in recent years since both the common and distinctive characteristics of chitosan share combined intelligence during the construction process on the basis of the aforementioned properties. In a word, chitosan has brought about brand-new opportunities for manifold anti-tumor therapies for decades, and new innovations are called for. 

### 3.2. Antimicrobial

**Figure 5:**
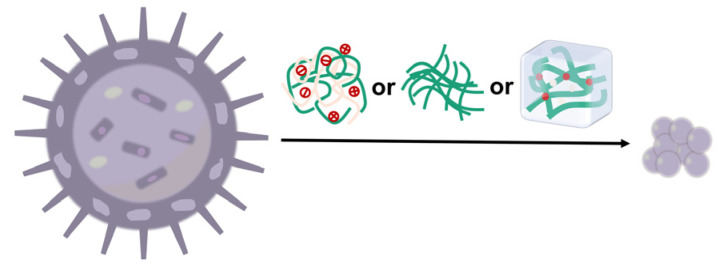
Chitosan and its derivative-related nano-biodevices for antimicrobial applications.

The antimicrobial ability, including antibacterial and anti-fungal, was illustrated in detail in Section 2.1.6 and Section 2.1.7, and for this reason there is provision of a congenital condition for chitosan to be involved in antimicrobial therapies. Compared with anti-tumor design, chitosan and its derivative-related nano-biodevices for antimicrobial therapies are more imaginative and dynamic, as presented in Figure 5. Meanwhile, the versatility of chitosan is well illustrated. 

On the basis of the staple antibacterial nature of chitosan, curcumin with excellent biological activity was conjugated onto carboxylated chitosan via an esterification reaction, and the obtained derivatives presented a better antibacterial effect on *Staphylococcus aureus* and *Escherichia coli* with mechanisms of scavenging free radicals and photodynamic antibacterial agents [92]. It is noteworthy that the design reversed the poor stability and insolubility of curcumin in water, overcoming the dilemmas in its application. This design is a simple sample of value concerning the fundamental modifiability and antibacterial nature of chitosan, but for further usage and realistic application, chitosan and its derivative-related nano-biodevice deeply correlate with skin wound closure and healing since skin trauma tends to be accompanied by bacterial infections. In a great deal of recent contributions, various hydrogels, such as injectable adhesive hydrogel, hydrogel as a sealant or wound dressing, and even shape memory cryogel, are extensively studied. For example, quaternary ammonium chitosan and tannic acid were facilely mixed under physiological conditions to fabricate an injectable hydrogel with injectability, self-healing ability, tissue-adhesiveness, broad-spectrum antibacterial ability, as well as rapid hemostatic capability [93]. In the hydrogel system, the ionic bonds and hydrogen bonds between quaternary ammonium chitosan and tannic acid were formed of the backbone, and sometimes the metal elements, such as Fe [94] and Ag [95], were incorporated for metal coordination bonds to endow hydrogel with more substantial and fulfilling natures. On one hand, the introduction of Fe (Ⅲ) (catechol-Fe) brought about excellent adhesiveness and an NIR-responsive photothermal property into the hydrogel [94]. On the other hand, a shape memory cryogel was prepared through the freeze–thaw cycle method [96] with a mixture of chitosan, citric acid, and tannic acid, as well as the important Ag nanoparticles. The resulted cryogel had good mechanical properties and interconnected microporous structures, and was thus capable of quickly absorbing a large amount of blood and promoting blood cell adhesion, compared with commercial gelatin sponges and gauze [95]. Similarly, for combating multidrug-resistant bacteria and treating skin abscesses, a novel chitosan-based antibacterial sponge was reported, which had a porous structure to absorb the wound exudate and stop continuous bleeding [97]. It is clear that chitosan-based antibacterial nano-biodevices have been exponentially studied, where hydrogel is an attractive form. In these engineered systems, the ionization-modified chitosan is frequently applied due to its attractive and available versatility, such as modifiability, biocompatibility, and ionic gelation ability. Indeed, chitosan is an ideal material for the fabrication of functional wound dressings with antibacterial properties and adhesion, hemostasis, anti-inflammatory, anti-oxidation, substance delivery, self-healing, stimulus response, conductivity, and wound monitoring features [98]. 

As discussed above, the practical applications of the antibacterial properties are integrated with trauma repair and healing, as well as skin abscesses. In most cases, the wound is an essential contributor to infection, and this concept could be extended to tissue regenerations, where the design strategies might be more intelligent. The chitosan matrix was able to load bioactive mucus extracts to fabricate porous scaffolds for hard tissue regeneration, with maintained in vitro bioactivity for bone and cartilage regeneration [99]. Additionally, in a recent work, the chitosan-based nanocomposite hydrogel was prepared to timely and continuously supply oxygen to mesenchymal stem cells to overcome deficient oxygen before vascularization in tissue regeneration scaffolds [100]. The form and concept of the nanocomposite is common and widely used in the design of nano-biodevices, and carbon nanofiller-reinforced chitosan composites of inorganic material, such as graphene-based hydroxyapatite and organic chitosan for antibacterial properties in bone tissue generation, were proposed, which generated good performance [101]. It could be summarized that while the design is related to multifaceted applications, chitosan is taken into consideration first since it seems to be a solid and conservative choice. It also generates opportunities for multifunctionality. 

As well as being antibacterial, chitosan is applicable in anti-fungal treatments to a similar extent, and both are essential components of antimicrobial management. Inspiringly, simple modification of chitosan is capable of bringing about incremental anti-fungal activity [65,102]. If it is narrowed and focused, fungal keratitis, as one of the infectious ocular diseases, should be one of the representative conditions where chitosan is available as a treatment strategy. Actually, as for the disease itself, the key to obtaining an effective and ideal therapy of fungal keratitis is to overcome the tear film barrier and corneal barrier on the ocular surface, and a chitosan-based dual-functional drug delivery platform has been designed and validated to enhance drug corneal penetration by virtue of prolonging the residence time and opening the corneal epithelial tight junctions [103]. Here, chitosan was carefully modified according to its strong interaction with the mucin layer by physical adhesion and covalent bonding, benefiting the prolonged residence time and the positive charge, favoring the opening of corneal epithelial tight junctions. 

Antimicrobial treatment is no doubt a primary focus at the medical level due to its frequent co-existence with other disorders. As discussed above, chitosan appears to be more advantageous and preferable when faced with such complicated situations because its versatility creates unique and superior conditions for constructing multi-functional nano-biodevices for medical applications. Despite the many challenges, it deserves continued exploration. 

### 3.3. Mucosal Drug Delivery 

It is widely acknowledged that mucosal drug delivery systems serve as a promising non-invasive and painless therapeutic approach for both systemic and topical administration for the considerable applications of mucosa, such as buccal delivery, nasal delivery, ocular delivery, rectal delivery, and vaginal delivery [104]. Since the mucosal route could accommodate systemic and topical administration under different conditions, its distinctiveness is responsible for the endless pursuit of a multifunctional biomaterial for the construction of mucosal drug delivery systems. Suitably, chitosan fulfills this requirement, and the highly customizable features make it a decent and inspiring candidate. 

In this field, the muco-adhesiveness is the primary focus of the chitosan-based mucosal drug delivery system to be achieved. Carboxymethylation was first used to improve the water solubility and biological activity of native chitosan, and afterwards, N-acetyl-L-cysteine (NAC) was introduced into the nano-system for enhancing mucosal adhesion, thus extending the stay of drugs in the mucosal site and improving their availability [105]. In another investigation, the authors modeled on intestinal diseases, partly manifested as intestinal barrier destruction, and low-molecular-weight chitosan (LMWC) was used to attach bilirubin (BR), composing LMWC-BRNPs to retain it in the gastrointestinal tract for longer [106]. Here, the mucoadhesive property was achieved in part by the water-soluble LMWC, which was positively charged for the negatively charged mucin. 

Another priority of chitosan facilitating mucosal drug delivery systems is the capability of self-gelation. As a non-invasive drug administration route, mucosal delivery is known for its painless effects, particularly for systemic treatments, and for this reason the patch form is popular. For one thing, some tailored oral patches were elaborately fabricated for diverse usages, where mussel-inspired, chitosan-catechol adhesive patches (chitoral) were designed to enhance healing of oral ulcers [107], and a biodegradable buccal gel patch comprising chitosan as the mucoadhesive matrix and ionic liquids/deep eutectic solvent as the transport facilitator was reported to improve the systemic delivery of insulin [108]. Besides, orally administered insulin-loaded nanoparticles motivated by self-gelation via chitosan and aqueous soluble snail mucin were also prepared [109]. On the other hand, the drug-loaded in situ bulk hydrogel within the nasal cavity for nasal mucosal drug delivery is further strong proof of its versatility [110].

Although it is attractive and engaging, its biodegradability is the cornerstone of all constructions, which is important for the biocompatibility and biological safety. This point is extremely fundamental and deserves more attention. While designing mucosal drug delivery systems, there appears to be numerous selections of specific materials. However, for most cases, the simplicity and versatility of the selected material is cautiously taken into consideration for the specificity of mucosal administration. Considerable works prefer chitosan and justify that chitosan is superior enough to construct multi-functional biomedical nanodevices for mucosal drug delivery. 

### 3.4. Oral Delivery

While injection administration remains prominent in efficient delivery of therapeutic agents, the non-invasive route is highly desired and attractive considering the virtue of convenience and patient compliance, in which case the oral delivery route tends to be more common and convenient compared to others. As a user-friendly application, the complexity of the implementation approach is indeed compulsory for the ease of use, which implies that there are some issues to be addressed while designing oral drug delivery nano-biodevices. Typically, unique physiological barriers, such as the mucosal barrier, cellular barrier, and systemic circulation, are dominant factors to be considered, and a chitosan-based nanoparticle for oral delivery of metformin for the typical chronic disease polycystic kidney disease (PKD) was prepared by virtue of the mild ionotropic gelation process, which is more favorable for chronic diseases compared to parenteral administration [111]. 

Oral delivery is suitable for both small-molecule drugs and large-molecule peptide/protein drugs if the focus is to overcome physiological barriers to achieve satisfactory oral bioavailability. For instance, a functionalized chitosan nanoparticle was designed as an efficient platform for oral delivery of paclitaxel [112], vitexin [113], and diclofenac [114], under which circumstances chitosan, adhering to the epithelial surface and interacting with mucin through strong electrostatic interactions, enhanced the materials’ residence time and drug concentration at the intestinal absorption site. When it comes to some bioactive components of large-molecule, including protein, drugs and antibodies, oral administration, as a non-invasive method, appears to be of particular value for its ability to circumvent some of the inconveniences of injections, and the oral delivery of this category of therapeutic agents is always beneficial. Ample works have focused on enhancing oral insulin [115] and antibody bioavailability [116], and nanocomposites with chitosan coatings are common and promising forms, where the enhanced oral delivery efficiency is also dependent on the gastrointestinal tract stability of agents beneficial for the protection effects of chitosan. 

### 3.5. Transdermal Drug Delivery

In the search for non-invasive and painless treatment modalities, transdermal drug delivery undoubtedly occupies an important role owing to its encouraging patient compliance, and it is more attractive than the mucosal delivery route in the aspect of ease of administration. For decades, transdermal drug delivery has been considered in topical treatment strategies, with forms of semi-solid formulations, patches, or microneedles. Recently, a chitosan-coated nanostructured lipid carrier was reported to precisely localize the drug in the breast for breast cancer treatment, and chitosan provided beneficial and compatible properties, mainly including bio-adhesiveness for skin deposition and penetration enhancement [117]. This type of physically targeted therapy derives benefits from transdermal delivery, and the skin permeability effectiveness is the prior obstacle to surmount comparative to other non-invasive drug delivery routes, which presents both opportunities and challenges. Usually, the choice of the transdermal route depends on the kind of drug and features of the disease. For glipizide, belonging to the biopharmaceutical classification system class II, chitosan-coated deformable liposomes were confirmed to ensure its stability, sustained release, prolonged residence time at the site of drug absorption, and increased permeability, which was of specific significance to glipizide with its short elimination half-life [118]. Besides, chitosan-based transdermal delivery systems could also be widely used in treatments for skin cancers, including squamous cell carcinoma, basal cell carcinoma, and melanoma, and chitosan with proper properties is well matched [119,120,121]. In conclusion, the success of chitosan as a transdermal drug carrier is based on the critical interplay of its integrated natures.

**Table 1:** Summary of biomedical applications of functional chitosan and its derivative-related nano-biodevices.

Biomedical Applications	Examples of Nano-Biodevices	Available Multi-Properties for Design	Refs.
Anti-tumor	DOX-encapsulated polymeric nanoparticle surface-decorated with chitosan	(1) Chemical modifiability for access to a wide range of features (2) Cationic nature for endosomal escape (3) Water solubility and crosslinking ability, favoring the form of injectable hydrogel (4) Biodegradability for in vivo safety (5) Special targeting ability	[87]
	DOX-loaded and aptamer-mesoporous silica nanoparticle (MSN) bioconjugates coated with chitosan (Dox-Chi-MSNs)		[88]
	In situ injectable nanocomposite hydrogel		[89]
	Glycol chitosan nanoparticles		[91]
Antimicrobial	Injectable quaternary ammonium chitosan (QCS)/tannic acid (TA) hydrogel	(1) Natural broad-spectrum antimicrobial activity (2) Biodegradability for in vivo safety (3) Structural modifiability for extended functionality (4) Adhesive property (5) Film-forming property (6) Water solubility and crosslinking ability, favoring the form of hydrogel	[93]
	Chitosan (CS)/citric acid (CA)/silver (Ag) cryogel		[95]
	CS-ZnO (zinc oxide particles)/Ce6 (chlorin) sponge		[97]
	Chitosan-based dual-functional platform for ocular topical delivery of econazole		[103]
Mucosal drug delivery	Mannose-anchored quaternized chitosan/thiolated carboxymethyl chitosan composite nanoparticles (M-N-2-HACC/BSA/N-CMCS NPs)	(1) Structural modifiability for extended functionality (2) Water solubility and crosslinking ability, favoring the form of hydrogel (3) Adhesive property (4) Film-forming property (5) Biocompatibility	[105]
	Bilirubin (BR)-attached low-molecular-weight, water-soluble chitosan nanoparticles (LMWC-BRNPs)		[106]
	Mussel-inspired, chitosan-catechol adhesive patches (Chitoral)		[107]
	In situ gelling starch nanoparticle (SNP)/O-carboxymethyl chitosan (CMCh) nanoparticle network hydrogel		[110]
Oral delivery	Polylysine and poly(lactide) co-modified thiolated chitosan/paclitaxel (PY-CS-PLA/PTX)	(1) Biocompatibility (2) Structural modifiability for extended functionality (3) Water solubility and accessible amphiphilicity for drug delivery	[112]
	Chitosan and insulin-loaded, zein-carboxymethylated short-chain amylose nanocomposites (IN-Z-CSA/CS)		[115]
Transdermal drug delivery	Transdermal glipizide delivery system based on chitosan-coated deformable liposomes (GLP-CS-DL1)	(1) Biocompatibility (2) Structural modifiability for extended functionality	[118]

## 4. Concluding Remarks

In most cases, the tendency to gravitate toward a particular material depends on the extent to which the material satisfies the design demands. As for biomedical applications, chitosan is known for its many excellent properties, because it is not restricted to a wide range of originations, and due to its low cost, biodegradability, and biocompatibility. In the current review, we first discussed recent advances in rationally constructing chitosan and its derivative-related nanodrug delivery system, mainly referring to two major areas concerning design and preparation. For material property-oriented design, the full scale of chitosan’s properties was presented, ranging from the number of amino groups, determined by molecular weight, water solubility, amphiphilicity, cationic nature, and immunogenicity, to its nucleus-targeting ability. Subsequently, in the Discussion Section, two major diseases, defined as microbial infection and inflammation, were analyzed, respectively. They are separated from each other but somehow have similarities and associations, as a result of which chitosan is widely used. The key for chitosan’s generation of antimicrobial activity is the disruption of the cell wall and membrane, with following entry effects. All of these characteristics interact with each other, generating an integrative effect. The emphasis on design cannot be overstated, and the importance of how to prepare is also self-evident, which was summarized in the following part on the preparation method. This is a process of forming a holistic view of the carrier material and encapsulated therapeutic agents, and the solubility of agents appears to be pivotal. On the basis of the aforementioned factors, biomedical applications of functional chitosan and its derivative-related nano-biodevices were logically and naturally presented in the third section. The related biomedical treatments covered anti-tumor, antimicrobial, mucosal drug delivery, oral drug delivery, and transdermal drug delivery, where the features of diseases are in great correlation with chitosan’s properties, showing combined effects. Here, the introduced design demonstrated the versatility of chitosan well, and the concept of being simple yet effective was well illustrated and vividly represented. A simple and delicate design with appropriate and compliant application requires profound knowledge of the specific disease and material, where every attempt is worthwhile.

## 5. Future Perspectives

Nevertheless, there are still some points of concern regarding chitosan. For one thing, as for biosafety, it is of urgent necessity to pay more attention to chitosan of high molecular weight, especially in the treatment of specific diseases, which needs more attention to associate chitosan with the disease itself. Further, the control of impurities and quality in the industrial production of chitosan are equally worthy of attention. It is a pity that rare chitosan-based nano-biodevices have been commercialized, and thus the in vivo pharmacokinetic and pharmacokinetic processes remain ambiguous. This is brought on by a multitude of factors, and it deserves further exploration. 

## Data Availability

Not applicable.
